# Supplementary material for: Upconversion particle-assisted NIR polymerization enables microdomain gradient photopolymerization at inter-particulate length scale
Source: Nat Commun. 2023 Jun 20;14:3653. doi: 10.1038/s41467-023-39440-2 (PMC10282001; doi:10.1038/s41467-023-39440-2)
Supplement: Supplementary file 1 — Supplementary Information [file 41467_2023_39440_MOESM1_ESM.pdf]

Supplementary Information

Upconversion Particle-Assisted NIR Polymerization Enables Microdomain gradient Photopolymerization at Inter-particulate Length Scale

Peng Hu,[1] Hang Xu,[1] Yue Pan,[1] Xinxin Sang,[1,2] Ren Liu[1,2]*

[1]International Research Center for Photoresponsive Molecules and Materials, Jiangnan University, Jiangsu 214122, PR China.

[2]Key Laboratory of Synthetic and Biological Colloids, Ministry of Education, Jiangnan University, Jiangsu 214122, PR China.
Corresponding author. E-mail: liuren@jiangnan.edu.cn

This PDF file includes:

Supplementary Table 1
Supplementary Fig. 1 to Fig. 6

Supplementary Table 1. Data summary of mechanical properties of cured materials under different NIR irradiation time

Samples	Tensile strength (MPa)	Tensile Modulus (MPa)	Tensile toughness (MJ/m^3)
8 s	28.4 ± 3.9	655 ± 93	2.43 ± 0.21
15 s	45.8 ± 2.5	1198 ± 14	1.80 ± 0.1
25 s	57.2 ± 1.9	1471 ± 12	1.72 ± 0.13
50 s	69.6 ± 1.9	1615 ± 80	2.53 ± 0.15
100 s	66.4 ± 2.3	1788 ± 48	1.76 ± 0.18
UV	50.6 ± 4.8	942 ± 54	1.41 ± 0.12

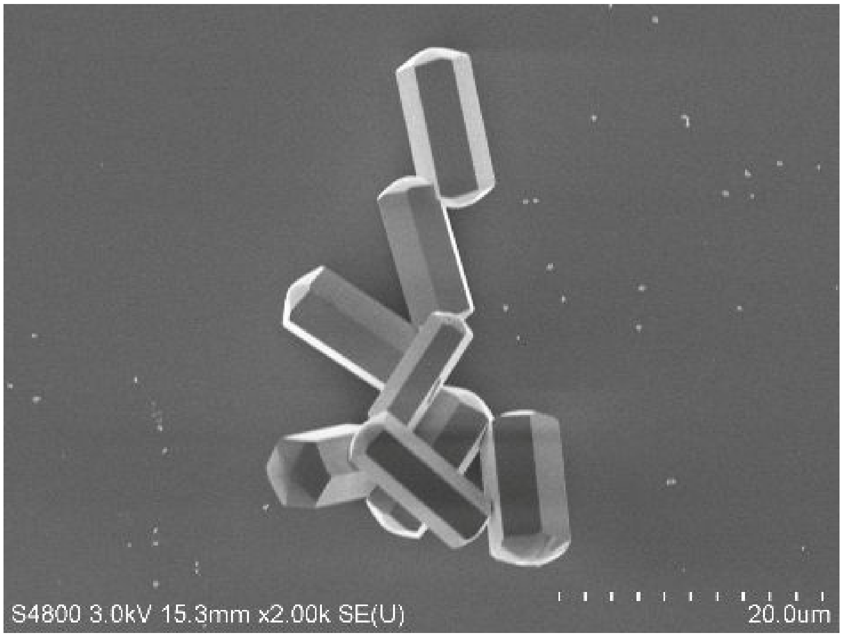

Supplementary Fig. 1 Morphology of upconvertion particles. SEM images of the UCPs used in this work. $NaYbF_4$: Tm (99.5 mol% Yb, 0.5 mol% Tm).

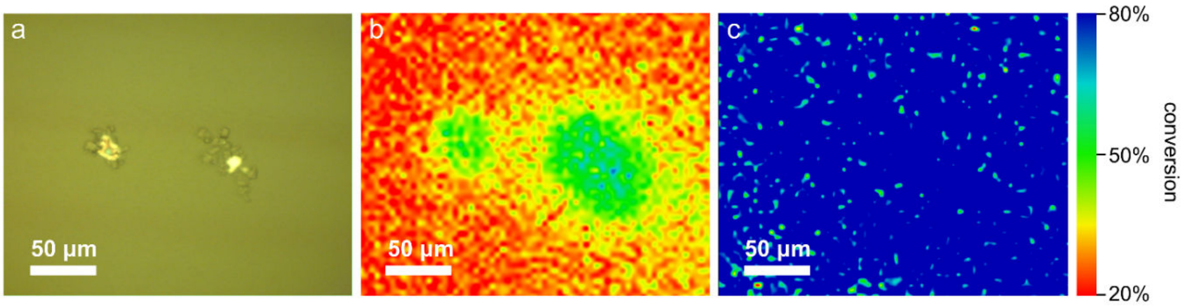

Supplementary Fig. 2 Homogeneous curing process of the material. **a** bright-field image of UCPs in curing system; **b** the distribution of double bond conversion and **c** after 60 s irradiation (25 W/cm^2, 0.5 wt% UCPs).

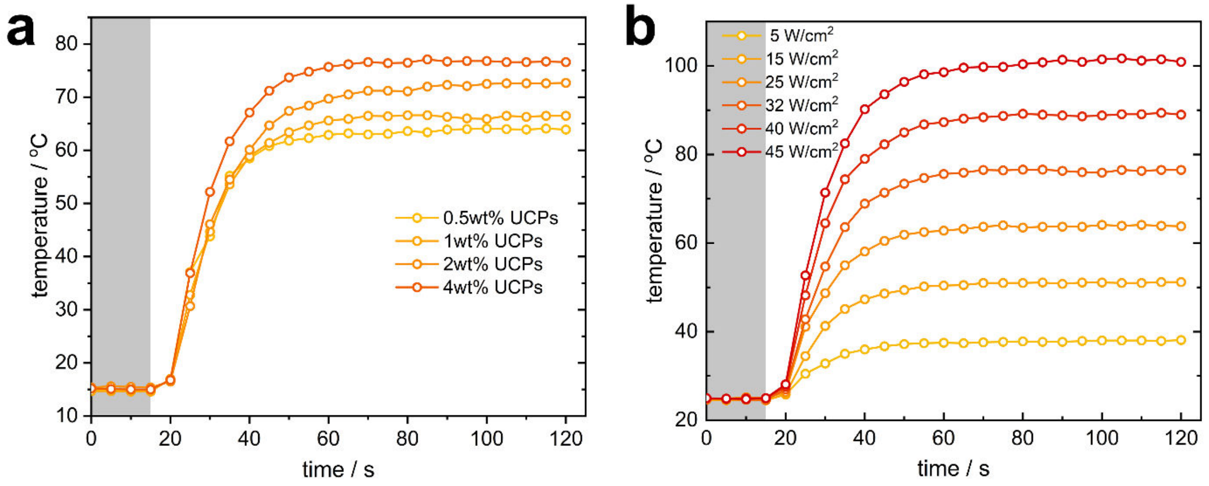

Supplementary Fig. 3 Thermal effects of the near-infrared light. **a** temperature evolution of monomers at different UCPs concentrations under near-infrared irradiation (25 W/cm^2, without initiator); **b** temperature evolution of monomers under different laser power irradiation (1 wt% UCPs, without initiator).

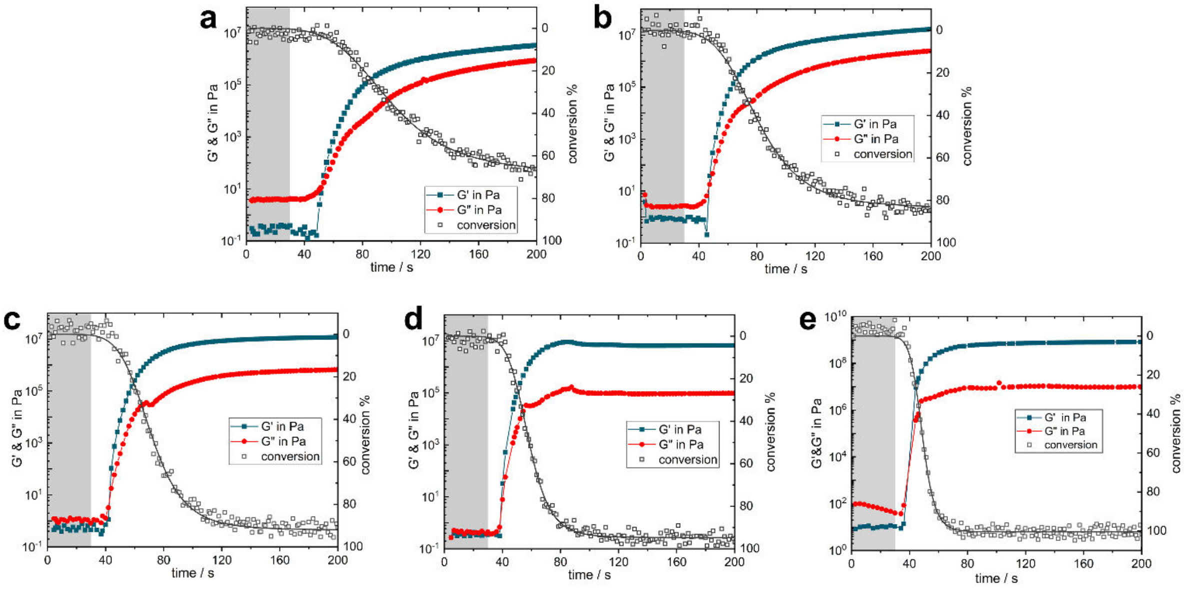

Supplementary Fig. 4 Gel behavior of UV photopolymerization. Detection of the gel point of UV curing systems at different temperatures, **a-e** for 10°C, 25°C, 40°C, 60°C and 80°C respectively. 1 wt% BAPO in BPA10EODMA, 8 mW/cm^2.

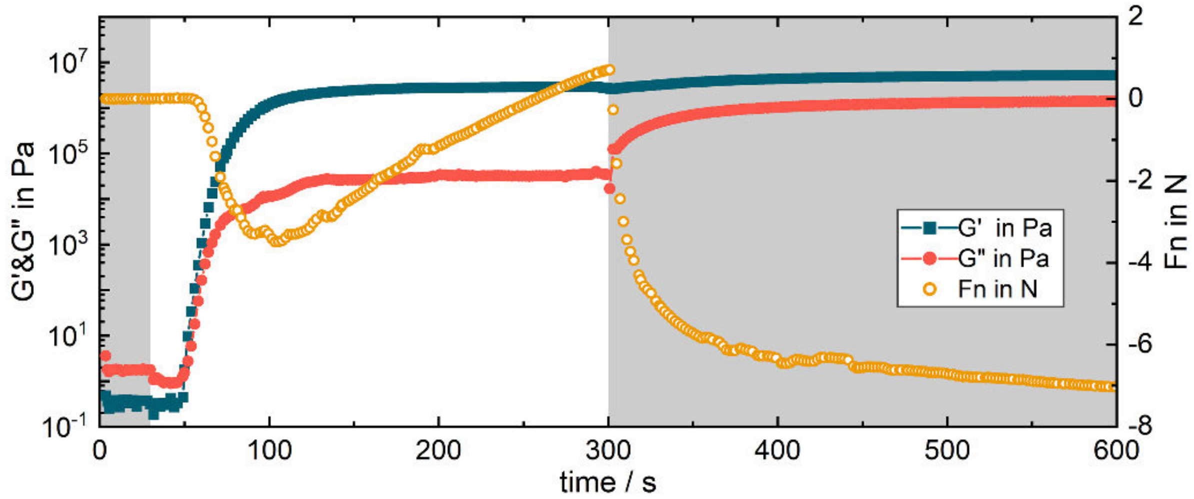

Supplementary Fig. 5 Testing for material shrinkage stress. Detection of the F_n of NIR curing systems. 1 wt% BAPO, 1 wt% UCPs in BPA10EODMA, 25 W/cm^2.

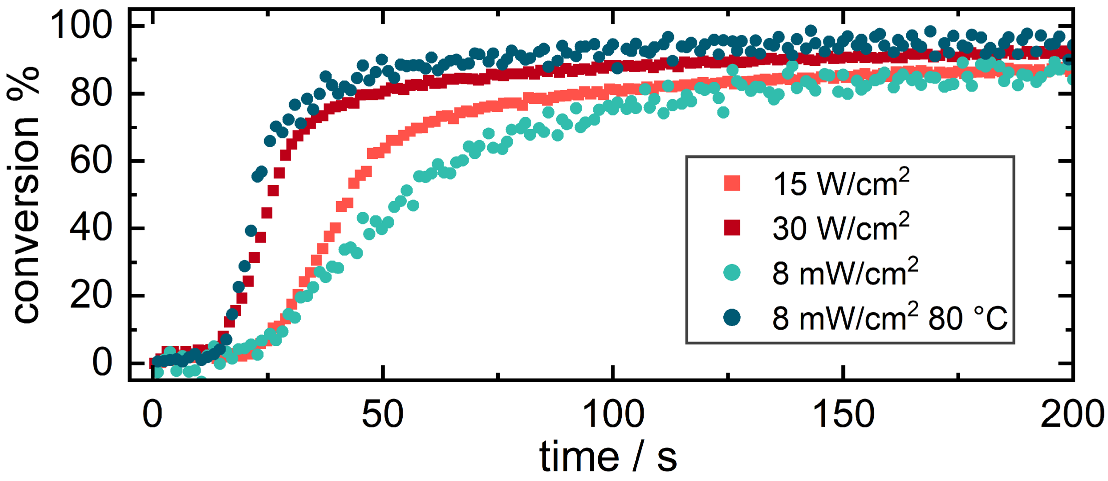

Supplementary Fig. 6 Kinetics of polymerization under different light sources irradiation. time-to-conversion profiles under different curing condition, the squares represent NIR and the dots represent UV, 1 wt% BAPO, 1 wt% UCPs in RY1101/TMPTA=7:3.
