# Supplementary material for: Upconversion particle-assisted NIR polymerization enables microdomain gradient photopolymerization at inter-particulate length scale
Source: Nat Commun. 2023 Jun 20;14:3653. doi: 10.1038/s41467-023-39440-2 (PMC10282001; doi:10.1038/s41467-023-39440-2)
Supplement: Supplementary file 3 — Description of Additional Supplementary Files [file 41467_2023_39440_MOESM3_ESM.pdf]

Description of Additional Supplementary Files

File Name: Supplementary Movie 1
Description: A 50 g sharp cone was used to impact the near-infrared and ultraviolet cured samples respectively (played at 5x speed).
